# Global landscape of early-onset thyroid cancer: current burden, temporal trend and future projections on the basis of GLOBOCAN 2022

**DOI:** 10.7189/jogh.15.04113

**Published:** 2025-04-11

**Authors:** Qianyun Jin, Jie Wu, Caiyun Huang, Jingjing Li, Yunmeng Zhang, Yuting Ji, Xiaomin Liu, Hongyuan Duan, Zhuowei Feng, Ya Liu, Yacong Zhang, Zhangyan Lyu, Lei Yang, Yubei Huang

**Affiliations:** 1 Key Laboratory of Molecular Cancer Epidemiology, Key Laboratory of Prevention and Control of Human Major Diseases, Ministry of Education, National Clinical Research Center for Cancer, Tianjin Medical University Cancer Institute and Hospital, Tianjin, China; 2 Key Laboratory of Carcinogenesis and Translational Research (Ministry of Education/Beijing), Beijing Office for Cancer Prevention and Control, Peking University Cancer Hospital & Institute, Beijing, China; 3 Peking University Cancer Hospital (Inner Mongolia Campus)/Affiliated Cancer Hospital of Inner Mongolia Medical University, Inner Mongolia Cancer Center, Hohhot, China

## Abstract

**Background:**

With rapid social-economic development and widespread screening, the surge in incidence and overdiagnosis of thyroid cancer is more worrying among the young than the general population. This problem, however, still lacks adequate attention.

**Methods:**

We retrieved the original data of current, past and future burden of thyroid cancer from the Global Cancer Observatory (GLOBOCAN) 2022. We calculated the age-specific mortality-to-incidence ratio (MIR) by dividing age-specific mortality rates by incidence rates to quantify potential overdiagnosis, and used Segi’s world standard population to calculate the age-standardised incidence rate (ASIR) and age-standardised mortality rate (ASMR). We then assessed the correlation between the human development index (HDI) and ASIR/ASMR using the linear correlation coefficient (r). Lastly, we characterised the temporal trend with the estimated annual percentage change (EAPC) and project the early-onset thyroid cancer burdens to 2050.

**Results:**

Globally, there were an estimated 239 362 (ASIR = 4.00 per 100 000 population) cases and 2409 (ASMR = 0.04 per 100 000 population) deaths from thyroid cancer among individuals aged <40 years in 2022. Compared to its ranking as the 7th most common cancer in the overall population, thyroid cancer rose to become the 2nd most common cancer among individuals <40 years. Nearly 99% of thyroid cancer cases in individuals <40 years of age (MIR = 0.01) may be potentially overdiagnosed, whereas 34% of cases in those >80 years (MIR = 0.66) were overdiagnosed. The ASIR of early-onset thyroid cancer varied widely (from 0.13 to 13.17 per 100 000 population), while the ASMR remains relatively similar and low across different regions. The ASIR of early-onset thyroid cancer increased with HDI (r = 0.69), while the ASMR decreased (r = −0.22). The ASIR of early-onset thyroid cancer had the fastest upward trend (EAPC in males vs. females: 9.88 vs. 9.28%) compared to early-onset cancers at other sites, while ASMR showed a downward trend (EAPC in males vs. females: −0.38% vs. −1.33%). The Republic of Korea experienced the highest EAPC for early-onset thyroid cancer ASIR (males vs. females: 29.95% vs. 23.04%). If national rates from 2022 remain stable, projected cases of early-onset thyroid cancer would decrease in high (−13.3%) and very high (−10.9%) HDI countries, but increase in low (96.5%) and medium HDI countries (11.7%).

**Conclusions:**

The trend of early-onset thyroid cancer at the global level is more alarming than that of thyroid cancer overall. The younger age at diagnosis of thyroid cancer, the higher risk of potential overdiagnosis. Without timely interventions, the thyroid cancer burden will inevitably become a serious global public health issue, especially for the young population.

Thyroid cancer is the most common malignancy of the endocrine system and the fifth most common cancer worldwide among females aged 20–84 years [1,2]. In 2020, there were an estimated 586 202 cases and 43 646 deaths from thyroid cancer, making it the 9th most prevalent cancer globally [3]. Since the 1980s, its incidence has been continuously increasing in most countries over the past three decades [1,4,5]. However, mortality concurrently remained low and stable or showed a declining trend during the same period [6]. The disparity in trends not only suggested that the increase in thyroid cancer is largely due to enhanced detection through organised or opportunistic screening, but also reflected the growing global trend of overdiagnosis, particularly in papillary carcinomas [1,4,7]. Moreover, with rapid socioeconomic development and widespread screening, the surge in incidence and overdiagnosis of thyroid cancer is expected to be more worrying among the young population than in the general population [4,8,9]. However, little attention has been paid to this worrying trend.

Alongside the growing introduction of modern screening/diagnostic techniques, previous studies found that higher thyroid cancer incidence positively correlated with the increase in the human development index (HDI) [4,10]. This link would largely reflect the prevalence of risk factors associated with socioeconomic development. Although most known risk determinants for thyroid cancer are non-modifiable [11], epidemiological studies have suggested that a substantial proportion of thyroid cancer may be attributable to suspected modifiable risk factors, including overweight or obesity, smoking, exposure to ionising radiation, maternal diabetes, maternal thyroid dysfunctions, and unhealthy diet [12-19]. However, fewer studies have investigated the global relationship between early-onset thyroid cancer and HDI, as well as possible geographic differences due to variations in exposure to modifiable risk factors across different countries.

Given this rapid growth in cases and issues with overdiagnosis, it is crucial to determine the global burden of this disease in order to inform local cancer prevention strategies in a timely manner. We therefore aimed to investigate and highlight the current global burden of early-onset thyroid cancer by comparing it with the burden of thyroid cancer in the general population. Specifically, we sought to examine the burden of early-onset thyroid cancer across various countries or territories, age-specific overdiagnosis, and temporal trends over recent years, as well as to project the number of cases and deaths from early-onset thyroid cancer to 2050.

## METHODS

### Data sources

We retrieved all our data from the Global Cancer Observatory (GLOBOCAN) 2022. In detail, this database is formed from data of cancer cases and deaths released by national or subnational cancer registries and aggregated by the International Agency for Research on Cancer (IARC) under the World Health Organization (WHO) according to uniform standards [20]. The GLOBOCAN database provides estimated burden data of 36 cancer diseases across 185 countries or regions worldwide, including age-stratified and sex-stratified data, up to the year 2022 [21].

The original data of current, past and future burden of thyroid cancer were extracted from GLOBOCAN 2022 sub-databases (Cancer Today, Cancer Over Time, Cancer Tomorrow) according to the definition set by the International Classification of Tumor Diseases and Related Health Problems, 10th Revision (ICD-10), code C73 [22-24]. The latest estimated incidence and mortality were used for describing and comparing the current burden of thyroid cancer globally. High-quality data derived from national or subnational cancer registries with continuous monitoring, including data submitted to the Cancer Incidence in Five Continents (CI5 plus) and the WHO mortality, and were used to present temporal trends in cancer-specific incidence and mortality. The projected new cases and deaths from thyroid cancer in 2050 were extracted from Cancer Tomorrow database and used to present future cancer burden based on estimated data in 2022.

We did not obtain ethical approval or patient consent for our study, as the data we used in our analysis were publicly available. We reported our findings following the STROBE guidelines [25].

### World regions and HDI

We presented the regional incidence and mortality according to the 21 aggregated world regions defined by the United Nations (UN) Population Division [26]. To illustrate the differences in thyroid cancer burden across countries with varying socioeconomic development levels, the country-specific information of HDI was extracted from the GLOBOCAN database, which is calculated based on the average years of education, expected years of education, life expectancy at birth and gross national income per capita according to the UN Development Program's Human Development Report 2021–22 [27]. All countries were reclassified into predefined four-tier HDI: low HDI (<0.550), moderate HDI (0.550–0.699), high HDI (0.700–0.799), and very high HDI (>0.8) [28]. Moreover, we used binary proxies of human development (transitioning *vs.* transitioned HDI) as synonyms for low and medium HDI *vs.* high and very high HDI, respectively.

### Statistical analysis

We used descriptive analysis statistics to present the incidence and mortality of early-onset thyroid cancer. We calculated age-standardised incidence rate (ASIR) and mortality rate (ASMR) per 100 000 population based on the 1966 Segi-Doll world standard population to remove the impact of different age structure on comparison across population [29,30] and used rank change chart of thyroid cancer incidence (or mortality) to present the differences in cancer-specific burden between individuals under 40 years old and the general population [31,32]. For countries that lacked their own cancer registries and mortality registry data, we used mortality-incidence-ratios sourced from neighbouring countries' cancer registry data to project the respective incidence and mortality rates, or simply adopted the rates of those countries or the region in general [26]. Given the substantial variation in population size among world regions and individual countries, we calculated standard errors (SE) of the ASR (including ASIR and ASMR) using the standard deviation of the sample rate from a binomial distribution.



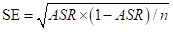



According to previous studies, we defined overdiagnosis as a condition that is diagnosed but would not lead to symptoms or death [33]. Unlike necessary medical diagnoses, cancer overdiagnosis refers to cancer that either does not progress or progresses so slowly that the patient dies from other causes before the cancer becomes symptomatic [33,34]. Previous studies suggested that overdiagnosis of thyroid cancer occurred primarily in the middle-aged patients [35]. Therefore, to investigate the overdiagnosis of thyroid cancer among different age groups, we determined the age-specific mortality-to-incidence ratio (MIR) by dividing the age-specific mortality rates with age-specific incidence rates, under the assumption that individuals within the same age group belonged to the same birth cohort [36]. Based on previous studies on young thyroid cancer (<40 years) and the peak age of thyroid cancer (around 40–50 years), we defined early-onset thyroid cancer as cases diagnosed at age <40 years [1,4,8]. We measured the correlation between HDI and ASIR/ASMR as linear correlation coefficient (*r*) and used the estimated annual percentage change (EAPC) as a metric to quantify temporal trends in ASIR/ASMR of early-onset thyroid cancer across entire available timeframe with continuous monitoring data [37-40]. After identifying the overlapping timeframe covered by continuous monitoring data across different countries, we recalculated the EAPC to improve comparability within the unified timeframe, including 2001–10 for ASIR and 2000–14 for ASMR. Finally, we estimated future cases and deaths from thyroid cancer of individuals aged under 40 years in 2050 based on demographic projections, assuming the national rates (including incidence/mortality rate and population growth rate) estimated in 2022 remain constant [3,26]. To present and facilitate the comparison of the burden of early-onset thyroid cancer between different genders, combined graphs of both genders were drawn instead of separate graphs.

We performed data analyses and visualisation using *R*, version 4.2.3 (R Core Team, Vienna, Austria) and Microsoft Excel, version 2016 (Microsoft Corporation, Redmond, WA, USA).

## RESULTS

### Global incidence and mortality of early-onset thyroid cancer in 2022

**Figure 1:**
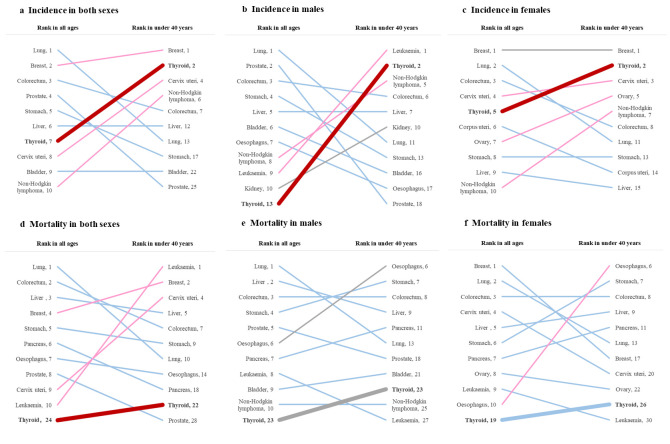
Global rank changes in incidence and mortality of selected top 10 common cancers in the total population and the young population under 40 years of age in 2022. Gray lines represent no change in rank. Pink and red lines represent increase in rank, and blue lines represent decrease in rank.

**Figure 2:**
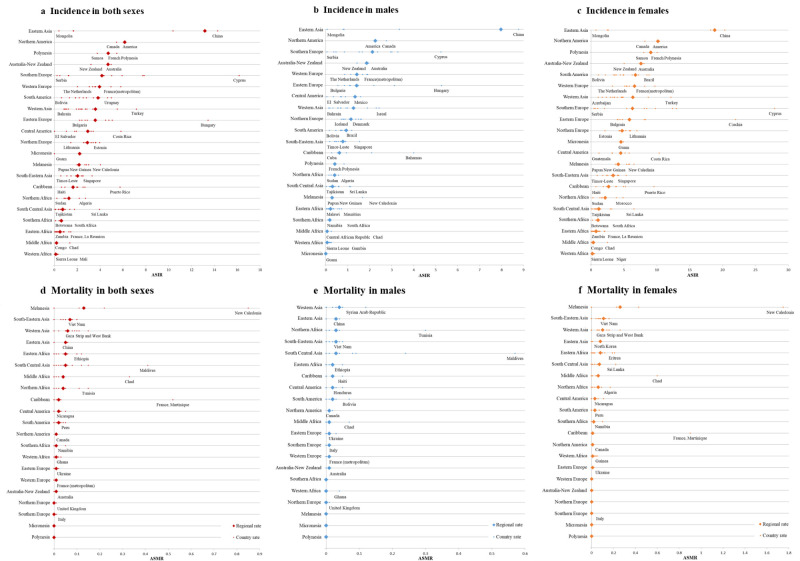
National ASIR and ASMR per 100 000 population of early-onset thyroid cancer grouped by UN-defined world regions in 2022. ASIR – age-standardised incidence rate, ASMR – age-standardised mortality rate.

Globally, an estimated 239 362 (males: 64 200; females: 175 162) new cases of early-onset thyroid cancer occurred in 2022, accounting for 15.71% (males: 11.01%; females: 18.63%) of all-site early-onset cancers (Figure S1 in the **Online Supplementary Document**). Compared to its ranking as the seventh most common cancer in the overall total population, thyroid cancer rose to become the second most common early-onset cancer among individuals under population <40 years, regardless of gender (**Figure 1**). The ASIR of early-onset thyroid cancer was 4.00 per 100 000 population worldwide, with females’ rates being approximately three times higher than males, at 6.10 and 2.10, respectively (Table S1 in the **Online Supplementary Document**). The ASIR of early-onset thyroid cancer per 100 000 population varied greatly across different regions, with the highest ASIR observed in Eastern Asia (13.17), followed by North America (6.16), Polynesia (4.72), and Australia-New Zealand (4.70). The lowest ASIR per 100 000 population was found in Western Africa (0.13), followed by Middle Africa (0.20) and Eastern Africa (0.50). Within Eastern Asia, China had the highest early-onset thyroid cancer ASIR (14.3 per 100 000 population), accounting for more than half of the incidence burden of early-onset thyroid cancer worldwide (**Figure 2**; Table S1 and S2 in the **Online Supplementary Document**).

In 2022, there were an estimated 2409 deaths (males: 743; females: 1666) from thyroid cancer before the age of 40 years worldwide (ASMR per 100 000 population for both sexes: 0.04; for males: 0.03; for females: 0.06), ranking it as the 22nd most common early-onset cancer according to mortality (23rd among males, 26th among females) of all-site early-onset cancers (**Figure 1**; Table S1 in the **Online Supplementary Document**). The early-onset thyroid cancer ASMR per 100 000 population was generally low and relatively similar across different regions, ranging from 0.00 (Southern Europe, Northern Europe, Micronesia, and Polynesia) to 0.13 (Melanesia) in 2022. Among Melanesia, New Caledonia has the highest ASMR of early-onset thyroid cancer, at 0.85 per 100 000 population (**Figure 2**; Table S1 and S2 in the **Online Supplementary Document**).

The MIR exhibited an upward trend with age at diagnosis (**Table 1**). Specifically, the lowest MIR of 0.01 was observed in the population <40 years, whereas the highest MIR of 0.66 was recorded among individuals aged ≥80 years. Namely, nearly 99% of thyroid cancer cases in individuals <40 years did not die from the disease and would be potentially overdiagnosed, while 34% of thyroid cancer were overdiagnosed for those >80 years of age. Nearly 99% of thyroid cancer cases in individuals under 40 may be potentially overdiagnosed, whereas 34% of cases in those over 80 were overdiagnosed.

**Table 1:** The global ASIR, ASMR, and MIR of thyroid cancer per 100 000 population by different age groups

Age in years	Number of cases	ASIR	Number of deaths	ASMR	MIR
0–39	239 399	4.03	2409	0.04	0.01
40–49	175 867	18.03	2817	0.29	0.02
50–59	214 165	24.81	6766	0.77	0.03
60–69	121 656	20.15	11 342	1.84	0.09
70–79	52 729	15.35	12 986	3.66	0.24
≥80	17 398	10.49	11 187	6.88	0.66

ASIR – age-standardised incidence rate, ASMR – age-standardised mortality rate, MIR – mortality-to-incidence ratio

### Link between thyroid cancer and HDI

In 2022, over 70% of early-onset thyroid cancer cases were diagnosed in high-HDI countries, corresponding to an ASIR of 8.24 per 100 000 population, while low-HDI countries had the lowest ASIR of 0.46. Likewise, 44.50% of deaths from thyroid cancer before age 40 years occurred in high-HDI countries, amounting to an ASMR of 0.05 per 100 000 population, while very high-HDI countries had the lowest ASMR at 0.01 (Table S1 in the **Online Supplementary Document**). Early-onset thyroid cancer ASIR positively increased with HDI (*r* for both sexes = 0.69; *r* for males = 0.68; *r* for females = 0.71), while there was a negative association between early-onset thyroid cancer ASMR and HDI (*r* for both sexes = −0.22; *r* for males = −0.23; *r* for females = −0.24) (Figure S2 in the **Online Supplementary Document**).

### Temporal trend of early-onset thyroid cancer incidence and mortality

**Figure 3:**
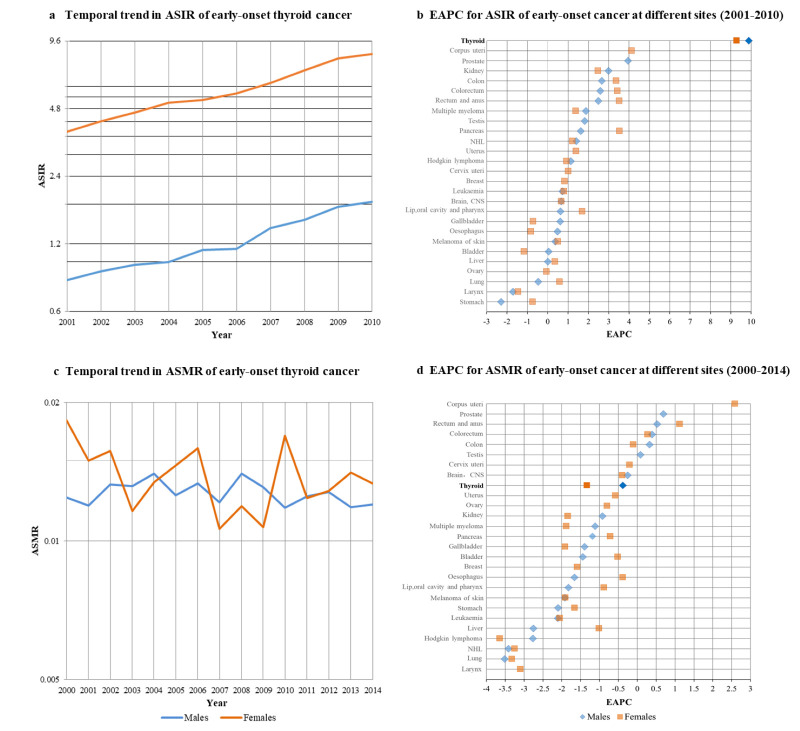
Global temporal trends in ASR per 100 000 population of early-onset thyroid cancer and other cancers on the basis of available data. ASR – age-standardised rate, ASIR – age-standardised incidence rate, ASMR – age-standardised mortality rate.

Early-onset thyroid cancer ASIR showed an increasing trend in both sexes in most countries (Figure S3, Panel A and Table S3 in the **Online Supplementary Document**) such as Republic of Korea (EAPC in males: 25.81%; females: 20.42%), Poland (males:11.70%; females:7.48%), and China (males: 10.16%; females: 6.24%). During the monitoring period from 2001 to 2010, the global EAPC for early-onset thyroid cancer ASIR were 9.88% and 9.28% for males and females. Comparing to EAPCs for ASIR of other early-onset cancers, thyroid cancer ranked as the most rapidly increasing early-onset cancer (**Figure 3**, Panels A and B; Tables S4 and S5 in the **Online Supplementary Document**), regardless of gender. Meanwhile, the highest national EAPC was observed in Republic of Korea (males: 29.95%, females: 23.04%), followed by Estonia (males: 26.30%, females: 0.86%), Turkey (males: 18.65%, females: 15.49%), China (males: 17.01%, females: 10.57%), and Costa Rica (males: 11.32%, females: 7.16%) (Figure S3, Panel B and Table S3 in the **Online Supplementary Document**).

Based on all available data with continuous monitoring, the ASMR of early-onset thyroid cancer showed a slightly declined trend in most countries (Figure S3, Panels C and D, Table S6 in the **Online Supplementary Document**). During the unified period (2000–14), the global EAPC for early-onset thyroid cancer ASMR were −0.38% in males and −1.33% in females (**Figure 3**, Panels C and D; Table S4 and S5 in the **Online Supplementary Document**). At the country level, EAPC for early-onset thyroid cancer ASMR in Republic of Korea was 0.76% in males and −3.35% in females. The lowest EAPCs for ASMR were −15.56% in Latvian males and −31.46% in Latvian females (Figure S3, Panel D and Table S6 in the **Online Supplementary Document**).

### Future projections of early-onset thyroid cancer

Based on the projected population growth and aging, and assuming that national rates in 2022 remain unchanged (Table S7 in the **Online Supplementary Document**), there would be an estimated 239.40 thousand cases and 2.68 thousand deaths from thyroid cancer among individuals aged <40 years in 2050 worldwide, corresponding to increase by 12.2% in cases and by 11.0% in deaths compared to estimates in 2022, respectively. Subgroup analyses showed a different future burden of early-onset thyroid cancer among countries with different HDI levels. Specifically, the estimated number of cases of early-onset thyroid cancer in 2050 would only decrease in high and very high HDI countries by −13.3% and −10.9%, respectively, while it would increase in low and medium HDI countries by 96.5% and 11.7%, respectively. Similarly, the estimated deaths from thyroid cancer before age 40 years would also increase in low and medium HDI countries (by 89.8% and 10.1%) but decreased in high and very high HDI countries (by −13.9% and −10.7%). Similar changes in the estimated burden of early-onset thyroid cancer would be occurred in both sexes (Figure S4 in the **Online Supplementary Document**).

## DISCUSSION

In this study, we have provided the most up-to-date burden of early-onset thyroid cancer for the year 2022. Consistently with previous studies [1,4,7,13,41-43], we found that the global burden of early-onset thyroid cancer mirrors that of the overall thyroid cancer, including the marked geographical difference in incidence, significantly heavier burden for females *vs.* males, obvious links with socioeconomic development, and unprecedented growth trends in incidence over other cancers. Our findings further highlight the severity of potential overdiagnosis in early-onset thyroid cancer compared to other cancers through various indexes, including changes in incidence ranking, mortality ranking, MIR, and EAPC. More worryingly, it seems that the burden of early-onset thyroid cancer is likely to be more serious than that of the overall thyroid cancer in the future due to socioeconomic development and population growth, particularly in transitioning countries rather than in transitioned countries. All these facts place great pressure on public health services, exacerbating the challenge of the management of this growing epidemic of early-onset thyroid cancer.

Consistent with the substantial increase in overall thyroid cancer observed previously [1,42], we observed a rapid increase in early-onset thyroid cancer in many countries over the past decades. Since the 1980s, the increase in overall thyroid cancer has been initially observed in transitioned countries and subsequently in transitioning countries [44,45], attributed to the broader application of improved screening/diagnostic techniques, albeit with geographical heterogeneity [41,46-48]. Meanwhile, thyroid cancer mortality has remained at low level or even declined in most countries. These contrasting epidemics of early-onset thyroid cancer and thyroid cancer in general highly reaffirms the widespread overdiagnosis of thyroid cancer. Although the main contributor to thyroid cancer (namely papillary tumours) typically would not cause symptoms or death [42], most patients still choose total thyroidectomy and other aggressive treatments to avert potential risk [49]. In 2003–07, the estimated overdiagnosis of overall thyroid-cancer cases in females were 90.0% in Republic of Korea, followed by approximately 70.0–80.0% in USA, France, and Australia; and nearly 50.0% in Japan, Nordic countries, England, and Scotland [47]. In 2009–12, the estimated overdiagnosis increased to 93.0% in Republic of Korea females [7], 77.3% of males and 83.1% of female in China [44,50].

Notably, our results suggest that nearly 99.0% of early-onset thyroid cancer cases in individuals under 40 years of age are likely to be overdiagnosed, which is much higher that the proportion of overdiagnosis reported in Indian female under 35 years (74.0%) [45]. Due to lack of thyroid cancer screening guideline for this population globally, the increase in early-onset thyroid cancer would largely attribute to the opportunistic screening among asymptomatic population or symptom-driven examination. The increased consciousness about health among young individuals [51] and voluntary engagement in thyroid ultrasound examination at an earlier age [52] will also collaboratively contribute to the escalation in the detection of early-stage thyroid cancer caused by opportunistic screening. To cope with the overwhelming overdiagnosis of thyroid cancer, the IARC and several countries have begun to modify the clinical practice guidelines [45,53], including exercising caution against asymptomatic screening and overtreatment of small nodules (<1 cm) for thyroid cancer, as well as active surveillance preferable for patients with low-risk papillary thyroid microcarcinoma [54]. Under this guideline, the EAPC of thyroid cancer’s ASIR in the Republic of Korea had declined significantly from 22.6% during the period of 1999–2011 and further to 12.6% in the subsequent period of 2011–16 [55]. Besides unnecessary diagnosis and therapy, individuals not only derive no clinical benefit from overdiagnosis, but also experience physical, psychological, or financial harms [33,56]. To mitigate this, greater focus is needed on high-risk populations, as well as further work on resource allocation, modification of terminology to better align with the underlying biology of cancer, and development of tools that could potentially identify overdiagnosis at the molecular level in individual tumours [56].

The marked geographic differences in early-onset thyroid cancer incidence and its obvious correlation with HDI likely largely reflect the varying prevalence of environmental exposures and healthcare access associated with socioeconomic development, as well as differences in genetic predispositions across countries [57-59]. Several studies have established the association between high body mass index (BMI) and increased risk of thyroid cancer [18,60], especially in females [61]. The IARC has also concluded that there are sufficient evidence supporting the increased risk with higher BMI, with relative risk of 1.1 per increase of 5 BMI units [62]. The global age-standardised prevalence of obesity increased from 8.8% to 18.5% for females and from 4.7% to 14.0% for males in 1990–2022 [63]. Meanwhile, relatively more obvious increases in prevalence of obesity were observed in children and adolescents aged 5–19 years (0.7–5.6% for girls; 0.9–7.8% for boys) in 1975–2016 [64]. The ASIR of the 12 obesity-related cancers (including thyroid cancer) increased annually by 3.6% in China, with the largest rise observed among younger adults – ranging from 15.28% for those aged 25–29 years to 1.55% for those aged 60–64 years [65]. Given the persistent upward trend in socioeconomic development across numerous countries, coupled with increased exposure to overweight and obesity in the foreseeable future, we hypothesise that promoting healthy weight and avoiding overweight or obesity in young population would be the least costly intervention to prevent early-onset thyroid cancer.

Exposure to ionising radiation at young age is a well-known modifiable risk factor for the development of thyroid cancer, especially for papillary thyroid cancer [11,66]. Excess risk of thyroid cancer with childhood exposure decreased with age at exposure and years since exposure, and it would last for more than 50 years or even a lifetime [67,68]. All types of ionising radiation, whether natural and artificial sources, have been classified as group 1 carcinogen by the IARC [69]. During the past few decades, exposure to ionising radiation has gradually shifted from natural to artificial sources [70], while effective radiation dose per individual in general population has increased about 70% (from 3.6 mSv to 6.2 mSv) in the USA during 1980–2006 [71]. Currently, medical imaging procedures represented the most significant artificial source of ionising radiation, and they are necessary in most medical settings [71]. Although the radiation dose for such medical procedures has decreased as technology improved, the cumulative effective dose in general population will likely continue to increase in the future due to the introduction of new procedures and frequent exposures [70]. Therefore, national and international efforts are urgently needed to reduce radiation-related early-onset thyroid cancer. These efforts should include issuing policies and management strategies to minimise ionising radiation from medical diagnostic procedures, standardising medical diagnostic procedures for young population, raising awareness among healthcare professionals about radiation risks, and strictly controlling unnecessary imaging procedures [72,73].

Another important factor would be the increased uptake of radioactive iodine in the young population with iodine deficiency [74]. Both iodine deficiency and supplementation may modify this risk [75], with iodine intake exhibiting a U-shape relationship with the risk of thyroid disease [76]. An estimated 30% of the world's population is at risk of iodine deficiency [77]. One study found that thyroid cancer incidence has steadily increased after the implementation of iodine supplementation in Shanghai, China [78], while another showed that long-term iodine intake would led to excessive secretion of thyroid stimulating hormone and then cause significant proliferation of thyroid follicular epithelial cells, goitre, and would finally develop into thyroid cancer [79]. Iodine intake also correlated with the pathological subtypes of thyroid cancer, including high papillary cancer incidence in iodine-rich areas and follicular cancer in iodine-deficient areas [51]. Therefore, the stable, effective, and lowest possible dose of iodine supplementation should be recommended to reduce the risk of early-onset thyroid cancer in patients with iodine deficiency.

The gender disparities in thyroid cancer incidence and mortality highlighted the influence of sex hormones, genetics, and behavioural and social factors on the thyroid cancer burden [80-82]. Abnormal oestrogen level of females would partly contribute to higher thyroid cancer incidence in females *vs.* males [83]. Thyroid ultrasound examinations during reproductive age further increased the detection of female early-onset thyroid cancer [47]. Genetic disparities between men and women also play a role in the gender gap observed across countries, although there is currently a lack of compelling evidence supporting gender-specific genetic differences [57-59]. Lifestyle factors, such as smoking, alcohol consumption, and physical activity, may further amplify these gender disparities. A recent study in China revealed that the cancer burden linked to 23 modifiable risk factors is notably higher among men (51.2%) than women (34.9%) [84]. Another cross-sectional study of 156 887 Chinese patients found that men had significantly longer duration of hospitalization and higher total medical expenses per inpatient compared to women, highlighting gender disparities in healthcare access [85]. Furthermore, the interactions between these factors likely exacerbate the gender disparities [42,81]. A deeper understanding of the molecular mechanisms underlying gender disparities could offer valuable insights for developing sex-specific, targeted, and effective strategies for thyroid cancer prevention.

Beyond these findings, several limitations deserve attention regarding our study. First, a lack of histopathological characteristics limited us from effectively exploring the different subtypes epidemic of early-onset thyroid cancer and further investigation of overdiagnosis. Second, the unavailability of data on diagnostic sources complicated our determining of whether early-onset thyroid cancer detection is incidental or symptom-based diagnosis. Third, differences in data reliability and completeness across countries, particularly in low- and middle-income countries, may have affected the accuracy of cancer incidence and mortality, potentially introducing bias into the analysis. Fourthly, the absence of long-term, high-quality, and continuous monitoring data, along with reliance on estimated rates derived from neighbouring countries or regions, inevitably introduces bias and even underestimate the temporal trend and the global landscape of early-onset thyroid cancer. Moreover, changes in histopathological classification guidelines, along with variations in healthcare systems, diagnostic criteria, and practices across countries could have significantly impacted the applicability of reported early-onset thyroid cancer incidence. Additionally, due to gender disparities in thyroid cancer incidence and mortality and ensuing complex interactions, statistics description may undermine the potential insights into early-onset thyroid cancer risk profiles. Finally, assuming stability in rates from 2022 for future projections may be an oversimplification and may not fully account for factors such as demographic changes, advancements in screening practices, and variations in healthcare access. Future projections incorporating these factors are necessary to ensure more realistic predictions of future trends.

## CONCLUSIONS

Our study provides the most up-to-date overview of the burden of early-onset thyroid cancer globally, indicating that it is likely more alarming than that of thyroid cancer in general. We observed that the risk of overdiagnosis likely increases with younger age at diagnosis. National and international efforts, including exercising caution against asymptomatic screening, promoting a healthy weight, avoiding overweight or obese, as well as improving awareness and standardising the medical diagnostic procedures in young population, are urgently needed to limit overdiagnosis and curb the global growing epidemic of early-onset thyroid cancer. Otherwise, the early-onset thyroid cancer burden will inevitably become a serious global public health issue for health systems in many countries, especially those in the transitioning stage of development.

## Additional material


Online Supplementary Document
