# Supplementary material for: Global landscape of early-onset thyroid cancer: current burden, temporal trend and future projections on the basis of GLOBOCAN 2022
Source: J Glob Health. 2025 Apr 11;15:04113. doi: 10.7189/jogh.15.04113 (PMC11984623; doi:10.7189/jogh.15.04113)
Supplement: Online Supplementary Document [file jogh-15-04113-s001.pdf]

Supplement to: Jin QY, Wu J, Huang CY, Li JJ, Zhang YM, Ji YT, Liu XM, Duan HY, Feng ZW, Liu Y, Zhang YC, Lyu ZY, Yang L, Huang YB. Global landscape of early-onset thyroid cancer: current burden, temporal trend and future projections on the basis of GLOBOCAN 2022. J Glob Health. 2025;15:04113.

Supplementary Figure 1. Global proportions in estimated cases and deaths from cancers among population under 40 years in 2022.

Supplementary Figure 2. Link between HDI and ASR of early-onset thyroid cancer in 2022.

Note: HDI, Human Development Index. ASR, age-standardized rate per 100 000 population. ASIR, age-standardized incidence rate. ASMR, age-standardized mortality rate

Supplementary Figure 3. National EAPC for ASR of early-onset thyroid cancer on the basis of available data.

Note: EAPC, Estimated Annual Percentage Change. ASR, age-standardized rate per 100 000 population. ASIR, age-standardized incidence rate. ASMR, age-standardized mortality rate.

Supplementary Figure 4. Demographic projections of early-onset thyroid cancer cases and deaths (of thousands) up until 2050 based on estimates in 2022.

Note: HDI, Human Development Index.

Supplementary Table 1. Estimated burdens and ASR of early-onset thyroid cancer by world regions and HDI in 2022.

Note: HDI, Human Development Index. ASR, age-standardized rate per 100,000 population. ASIR, age-standardized incidence rate. ASMR, age-standardized mortality rate. SE, standard error.

Supplementary Table 2. National HDI and ASR of early-onset thyroid cancer in different countries in 2022.

Note: HDI, Human Development Index. ASR, age-standardized rate per 100,000 population. ASIR, age-standardized incidence rate. ASMR, age-standardized mortality rate. SE, standard error.

Supplementary Table 3. National EAPC for ASIR of early-onset thyroid cancer by sex during the available and unified periods.

Note: EAPC, Estimated Annual Percentage Change.

Supplementary Table 4. Global EAPC for ASIR and ASMR of early-onset cancer at different sites by sex during the unified period.

Note: EAPC, Estimated Annual Percentage Change.

Supplementary Table 5. Global ASIR and ASMR of early-onset thyroid cancer by sex during the unified period.

Note: ASIR, age-standardized incidence rate per 100,000 population. ASMR, age-standardized mortality rate per 100,000 population.

Supplementary Table 6. National EAPC for ASMR of early-onset thyroid cancer by sex during the available and unified periods.

Note: EAPC, Estimated Annual Percentage Change.

Supplementary Table 7. Projected cases and deaths (of thousands) from early-onset thyroid cancer by HDI and sex from 2022 to 2050.

Note: HDI, Human Development Index.

Supplementary Figure 1. Global proportions in estimated cases and deaths from cancers among population under 40 years in 2022.

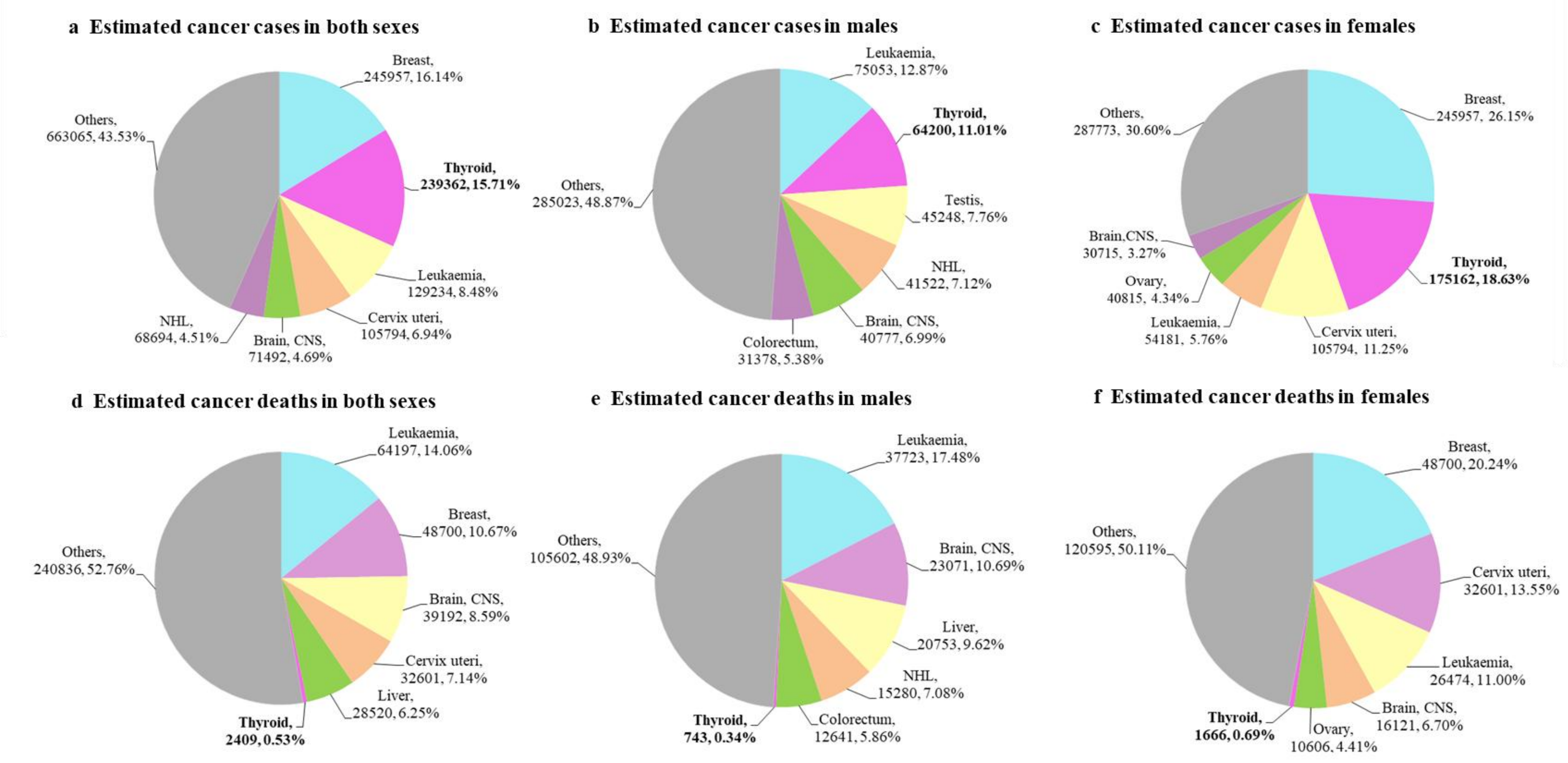

Supplementary Figure 2. Link between HDI and ASR of early-onset thyroid cancer in 2022.

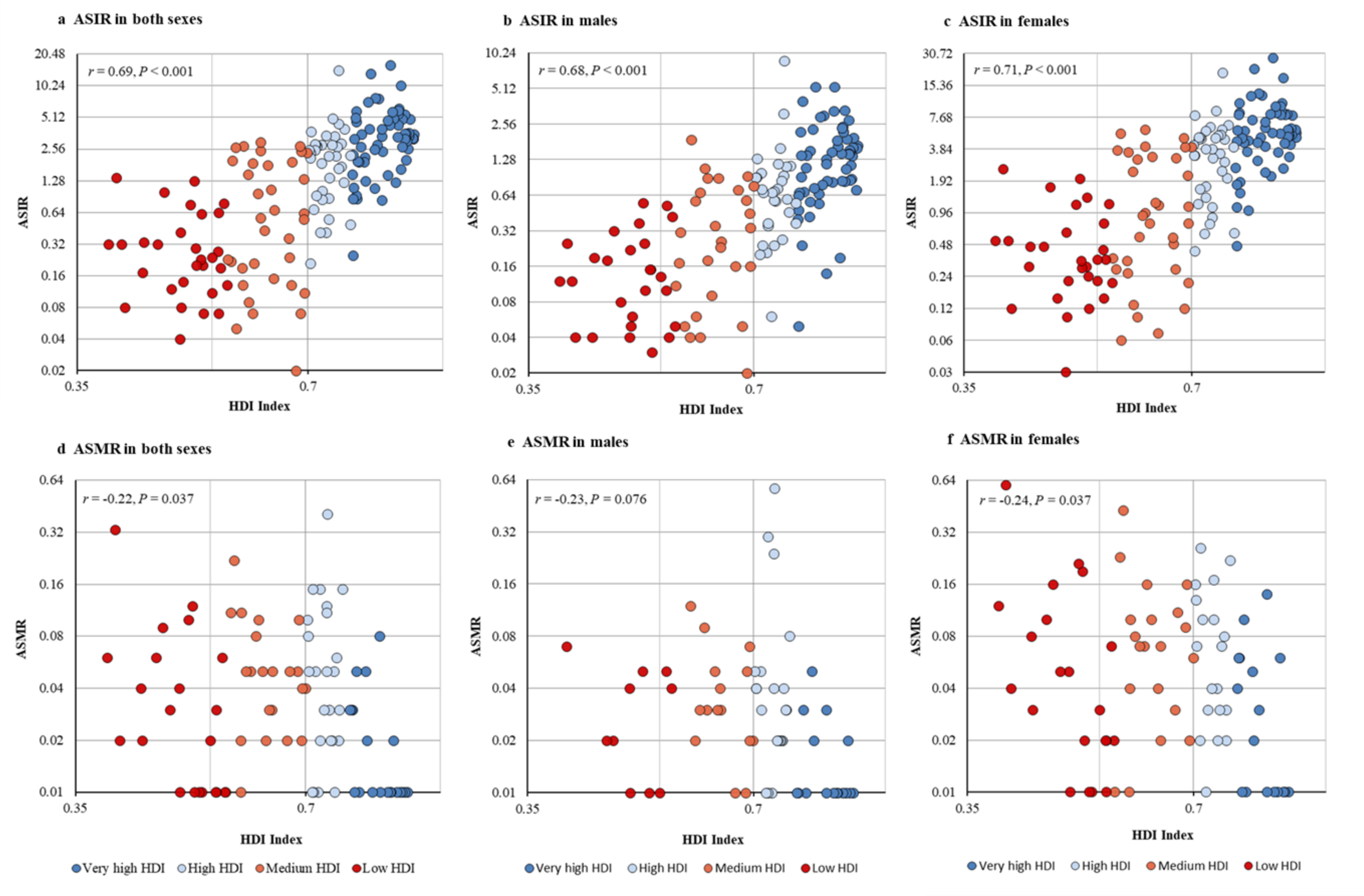

Note: HDI, Human Development Index. ASR, age-standardized rate per 100 000 population. ASIR, age-standardized incidence rate. ASMR, age-standardized mortality rate

Supplementary Figure 3. National EAPC for ASR of early-onset thyroid cancer on the basis of available data.

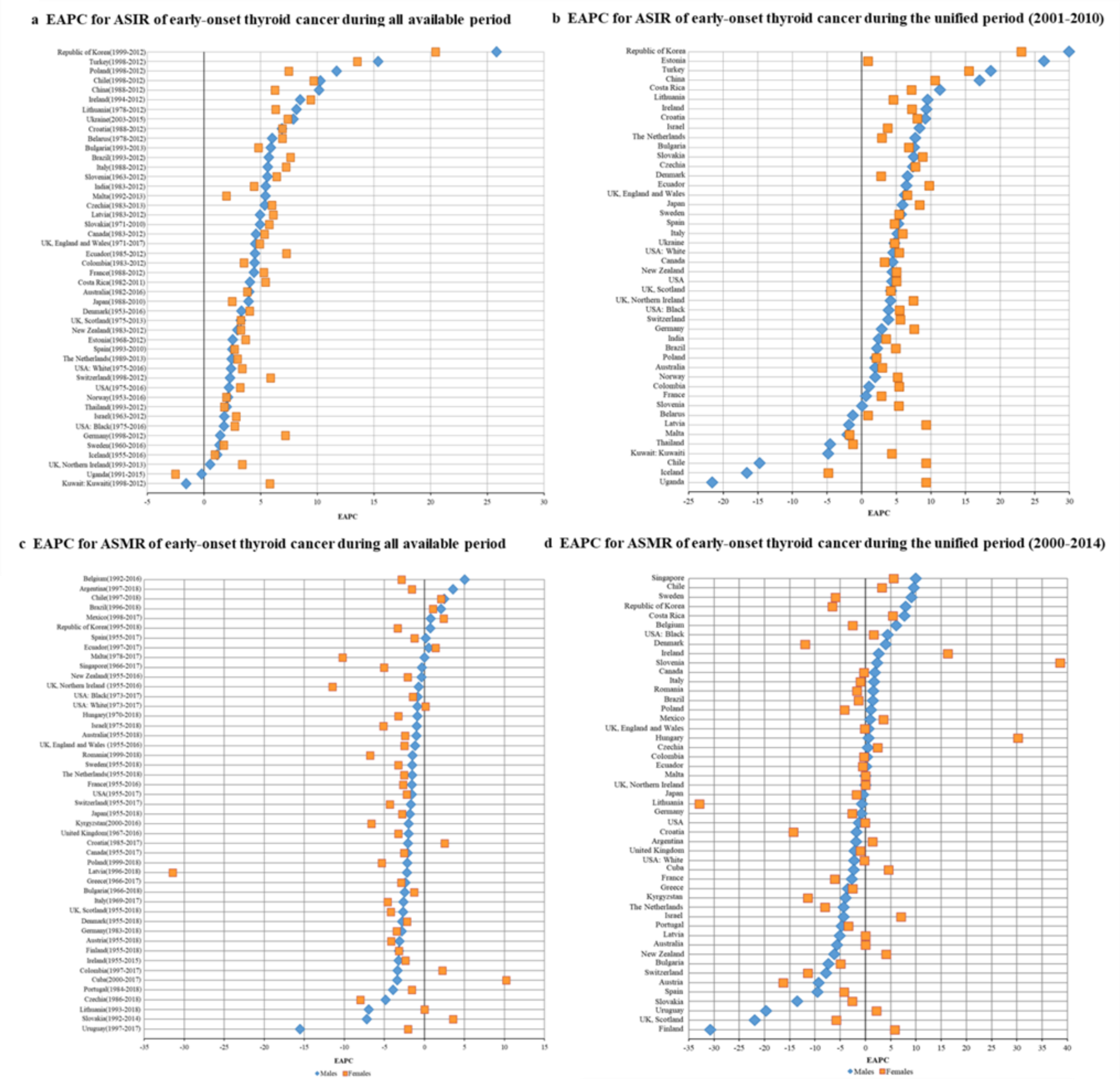

Note: EAPC, Estimated Annual Percentage Change. ASR, age-standardized rate per 100 000 population. ASIR, age-standardized incidence rate. ASMR, age-standardized mortality rate.

Supplementary Figure 4. Demographic projections of early-onset thyroid cancer cases and deaths (of thousands) up until 2050 based on estimates in 2022.

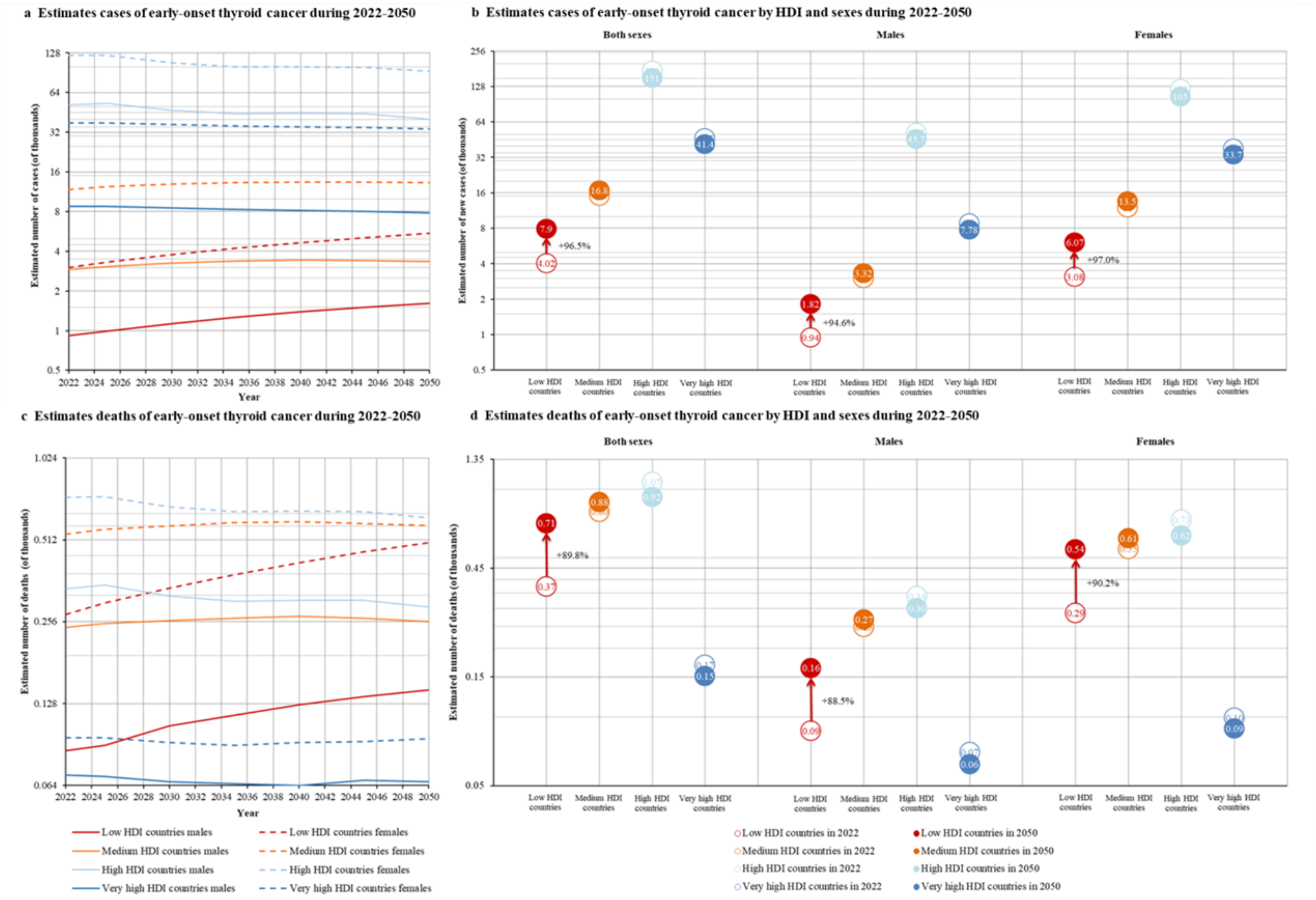

Note: HDI, Human Development Index.

Supplementary Table 1. Estimated burdens and ASR of early-onset thyroid cancer by world regions and HDI in 2022.

World regions/HDI	Incidence									Mortality					
	Both sexes			Males			Females			Both sexes		Males		Females	
	Cases(%)	ASIR	SE	Cases(%)	ASIR	SE	Cases(%)	ASIR	SE	Cases(%)	ASMR	Cases(%)	ASMR	Cases(%)	ASMR
World	239362(100)	4.00	-	64200(100)	2.10	-	175162(100)	6.10	-	2409(100)	0.04	743(100)	0.03	1666(100)	0.06
**Regions**															
Northern America	15261(6.73)	6.16	4.99E-07	2871(4.47)	2.25	4.20E-07	12390(7.07)	10.18	9.15E-07	32(1.33)	0.01	16(2.15)	0.01	16(0.96)	0.01
Eastern Asia	152249(63.60)	13.17	3.38E-07	47916(74.64)	7.97	3.64E-07	104333(59.55)	18.81	5.82E-07	607(25.20)	0.05	195(26.14)	0.03	412(24.73)	0.08
Eastern Africa	1762(0.74)	0.50	1.19E-07	376(0.95)	0.22	1.13E-07	1386(0.79)	0.79	2.12E-07	177(7.35)	0.05	28(3.77)	0.02	149(8.94)	0.08
Middle Africa	249(0.10)	0.20	1.27E-07	38(0.06)	0.06	9.73E-08	211(0.12)	0.35	2.41E-07	45(1.87)	0.04	6(0.81)	0.01	39(2.34)	0.06
Northern Africa	2457(1.03)	1.28	2.58E-07	388(0.60)	0.40	2.03E-07	2069(1.18)	2.17	4.77E-07	76(3.15)	0.04	25(3.36)	0.03	51(3.06)	0.06
Southern Africa	361(0.15)	0.62	3.26E-07	53(0.08)	0.18	2.47E-07	308(0.18)	1.07	6.10E-07	6(0.25)	0.01	1(0.13)	0	5(0.30)	0.02
Western Africa	373(0.16)	0.13	6.73E-08	83(0.13)	0.06	6.59E-08	290(0.17)	0.20	1.17E-07	24(1.00)	0.01	5(0.67)	0	19(1.14)	0.01
Caribbean	522(0.22)	1.64	7.18E-07	96(0.15)	0.62	6.33E-07	426(0.24)	2.67	1.29E-06	5(0.21)	0.02	3(0.40)	0.02	2(0.12)	0.01
Central America	4164(1.74)	2.94	4.56E-07	932(1.45)	1.33	4.36E-07	3232(1.84)	4.51	7.93E-07	37(1.54)	0.02	15(2.02)	0.02	22(1.32)	0.03
South-Eastern Asia	10570(4.42)	2.05	1.99E-07	2025(3.15)	0.78	1.73E-07	8545(4.88)	3.36	3.63E-07	343(14.24)	0.07	79(10.63)	0.03	264(15.85)	0.11
South Central Asia	11991(5.01)	0.73	6.67E-08	2533(3.95)	0.30	5.96E-08	9458(5.40)	1.19	1.22E-07	768(31.88)	0.05	263(35.40)	0.03	505(30.31)	0.07
Western Asia	8446(3.53)	3.60	3.92E-07	1603(2.50)	1.26	3.15E-07	6843(3.91)	6.35	7.68E-07	159(6.60)	0.06	49(6.59)	0.04	110(6.60)	0.10
Eastern Europe	6739(2.81)	3.58	4.36E-07	1318(2.05)	1.40	3.86E-07	5421(3.09)	5.83	7.92E-07	23(0.95)	0.01	13(1.75)	0.01	10(0.60)	0.01
Northern Europe	1986(0.83)	2.90	6.51E-07	401(0.62)	1.15	5.74E-07	1585(0.90)	4.71	1.18E-06	3(0.12)	0	2(0.27)	0	1(0.06)	0
Southern Europe	3501(1.46)	4.17	7.05E-07	899(1.40)	2.12	7.07E-07	2602(1.49)	6.31	1.24E-06	5(0.21)	0	3(0.40)	0.01	2(0.12)	0
Western Europe	4614(1.93)	3.95	5.82E-07	848(1.32)	1.41	4.84E-07	3766(2.15)	6.60	1.08E-06	7(0.29)	0.01	5(0.67)	0.01	2(0.12)	0
Australia-New Zealand	995(0.42)	4.70	1.49E-06	199(0.31)	1.86	1.32E-06	796(0.45)	7.59	2.69E-06	1(0.04)	0.01	1(0.13)	0.01	0(0.00)	0
Melanesia	187(0.08)	2.17	1.59E-06	13(0.02)	0.29	8.04E-07	174(0.10)	4.13	3.13E-06	11(0.46)	0.13	0(0.00)	0	11(0.66)	0.26
South America	12940(5.41)	3.83	3.37E-07	1606(2.50)	0.93	2.32E-07	11334(6.47)	6.75	6.34E-07	80(3.32)	0.02	34(4.58)	0.02	46(2.76)	0.03
Micronesia	9(0.00)	2.22	7.40E-06	0(0.00)	0	0	9(0.01)	4.53	1.51E-05	0(0.00)	0	0(0.00)	0	0(0.00)	0
Polynesia	23(0.01)	4.72	9.84E-06	1(0.00)	0.41	4.10E-06	22(0.01)	9.09	1.94E-05	0(0.00)	0	0(0.00)	0	0(0.00)	0
**HDI**															
Very high HDI countries	46494(19.42)	4.35	-	8799(13.71)	1.58	-	37695(21.52)	7.29	-	169(7.02)	0.01	70(9.42)	0.01	99(5.94)	0.02

High HDI countries	173833(72.62)	8.24	-	51468(80.17)	4.74	-	122365(69.86)	11.89	-	1072(44.50)	0.05	338(45.49)	0.03	734(44.06)	0.08
Medium HDI countries	15016(6.27)	0.83	-	2996(4.67)	0.32	-	12020(6.86)	1.38	-	795(33.00)	0.04	248(33.38)	0.03	547(32.83)	0.06
Low HDI countries	4019(1.68)	0.46	-	937(1.46)	0.21	-	3082(1.76)	0.71	-	373(15.48)	0.04	743(11.71)	0.02	286(17.17)	0.06

Note: HDI, Human Development Index. ASR, age-standardized rate per 100,000 population. ASIR, age-standardized incidence rate. ASMR, age-standardized mortality rate. SE, standard error.

Supplementary Table 2. National HDI and ASR of early-onset thyroid cancer in different countries in 2022.

Countries	HDI	HDI	ASIR						ASMR					
	Index	Index groups	Both sexes	SE	Males	SE	Females	SE	Both sexes	SE	Males	SE	Females	SE
Switzerland	0.962	Very high HDI	3.55	2.52E-06	1.67	2.44E-06	5.48	4.44E-06	-	-	-	-	-	-
Norway	0.961	Very high HDI	3.22	2.94E-06	1.59	2.86E-06	4.94	5.24E-06	-	-	-	-	-	-
Iceland	0.959	Very high HDI	3.23	1.14E-05	0.71	7.10E-06	5.86	2.21E-05	-	-	-	-	-	-
Australia	0.951	Very high HDI	4.97	1.67E-06	1.95	1.47E-06	8.06	3.01E-06	0.01	1.00E-07	0.01	1.00E-07	-	-
Denmark	0.948	Very high HDI	3.41	3.04E-06	1.61	2.94E-06	5.28	5.39E-06	-	-	-	-	-	-
Sweden	0.947	Very high HDI	3.64	2.37E-06	1.37	2.04E-06	6.02	4.36E-06	-	-	-	-	-	-
Ireland	0.945	Very high HDI	2.57	2.86E-06	0.88	2.35E-06	4.27	5.22E-06	-	-	-	-	-	-
Germany	0.942	Very high HDI	3.06	7.88E-07	1.14	6.63E-07	5.14	1.48E-06	0.01	5.00E-08	0.01	5.77E-08	-	-
The Netherlands	0.941	Very high HDI	2.03	1.40E-06	0.86	1.30E-06	3.25	2.53E-06	-	-	-	-	-	-
Finland	0.940	Very high HDI	3.60	3.25E-06	1.49	2.92E-06	5.83	5.92E-06	-	-	-	-	-	-
Singapore	0.939	Very high HDI	3.31	2.83E-06	1.54	2.68E-06	5.35	5.25E-06	-	-	-	-	-	-
Belgium	0.937	Very high HDI	3.61	2.25E-06	1.45	1.99E-06	5.84	4.08E-06	-	-	-	-	-	-
New Zealand	0.937	Very high HDI	3.21	3.13E-06	1.42	2.90E-06	5.03	5.59E-06	-	-	-	-	-	-
Canada	0.936	Very high HDI	5.44	1.46E-06	2.75	1.46E-06	8.20	2.56E-06	0.01	5.77E-08	0.02	1.41E-07	0.01	1.00E-07
Luxembourg	0.930	Very high HDI	4.96	1.06E-05	1.90	8.50E-06	8.17	1.98E-05	-	-	-	-	-	-
United Kingdom	0.929	Very high HDI	2.70	7.86E-07	1.06	6.91E-07	4.37	1.42E-06	0.01	5.77E-08	0.01	7.07E-08	-	-
Korea, Republic of Korea	0.925	Very high HDI	10.38	1.74E-06	3.35	1.35E-06	18.21	3.36E-06	-	-	-	-	0.01	1.00E-07
Japan	0.925	Very high HDI	1.65	5.18E-07	0.83	5.02E-07	2.51	9.23E-07	-	-	-	-	-	-
United States of America	0.921	Very high HDI	6.24	5.30E-07	2.20	4.39E-07	10.40	9.76E-07	0.01	1.86E-08	0.01	2.67E-08	0.01	2.58E-08
Israel	0.919	Very high HDI	5.51	3.07E-06	2.42	2.85E-06	8.70	5.49E-06	-	-	-	-	-	-
Malta	0.918	Very high HDI	5.94	1.36E-05	2.36	1.18E-05	9.80	2.53E-05	-	-	-	-	-	-
Slovenia	0.918	Very high HDI	4.34	6.08E-06	0.85	3.47E-06	8.07	1.20E-05	-	-	-	-	-	-
Austria	0.916	Very high HDI	3.36	2.41E-06	1.44	2.17E-06	5.38	4.38E-06	-	-	-	-		-
United Arab Emirates	0.911	Very high HDI	1.23	9.98E-07	0.19	4.15E-07	3.58	3.13E-06	0.02	1.15E-07	-	-	0.06	3.46E-07
Spain	0.905	Very high HDI	5.03	1.41E-06	1.66	1.15E-06	8.49	2.61E-06	0.01	7.07E-08	0.01	1.00E-07	0.01	1.00E-07
France (metropolitan)	0.903	Very high HDI	5.82	1.23E-06	1.87	9.87E-07	9.72	2.25E-06	0.01	5.77E-08	0.01	7.07E-08	-	-

Cyprus	0.896	Very high HDI	16.17	1.32E-05	5.24	1.03E-05	27.92	2.51E-05	-	-	-	-	-	-
Italy	0.895	Very high HDI	4.34	1.17E-06	3.28	1.42E-06	5.44	1.86E-06	0.01	5.77E-08	0.01	7.07E-08	0.01	1.00E-07
Estonia	0.890	Very high HDI	1.45	4.19E-06	0.82	4.10E-06	2.12	7.50E-06	-	-	-	-	-	-
Czechia	0.889	Very high HDI	4.79	2.81E-06	1.48	2.16E-06	8.27	5.29E-06	-	-	-	-	-	-
Poland	0.876	Very high HDI	2.42	1.02E-06	0.85	8.38E-07	4.08	1.89E-06	0.01	7.07E-08	0.01	1.00E-07	0.01	1.00E-07
Saudi Arabia	0.875	Very high HDI	3.38	1.06E-06	1.28	8.40E-07	6.14	2.19E-06	0.08	1.54E-07	0.03	1.13E-07	0.14	3.13E-07
Lithuania	0.875	Very high HDI	3.97	5.00E-06	1.17	3.70E-06	6.98	9.59E-06	-	-	-	-	-	-
Bahrain	0.875	Very high HDI	0.84	2.10E-06	0.14	8.08E-07	2.33	6.46E-06	-	-	-	-	-	-
Portugal	0.866	Very high HDI	7.74	3.76E-06	3.01	3.34E-06	12.38	6.69E-06	-	-	-	-	-	-
Latvia	0.863	Very high HDI	2.81	4.89E-06	0.78	3.49E-06	4.95	9.35E-06	-	-	-	-	-	-
Croatia	0.858	Very high HDI	7.86	5.75E-06	2.95	4.85E-06	12.98	1.06E-05	-	-	-	-	-	-
Chile	0.855	Very high HDI	2.53	1.35E-06	0.66	9.95E-07	4.45	2.54E-06	0.01	7.07E-08	-	-	0.03	2.12E-07
Qatar	0.855	Very high HDI	1.09	1.68E-06	0.54	1.27E-06	30.00	6.12E-06	-	-	-	-	-	-
Hungary	0.846	Very high HDI	13.45	4.94E-06	5.27	4.30E-06	22.00	9.06E-06	-	-	-	-	-	-
Argentina	0.842	Very high HDI	3.97	1.09E-06	0.74	6.57E-07	7.23	2.08E-06	0.02	8.16E-08	0.02	1.15E-07	0.02	1.15E-07
Türkiye	0.838	Very high HDI	7.18	1.06E-06	2.30	8.40E-07	12.16	1.96E-06	0.05	8.57E-08	0.05	1.21E-07	0.05	1.21E-07
Montenegro	0.832	Very high HDI	1.27	5.68E-06	1.52	8.78E-06	1.01	7.14E-06	-	-	-	-	-	-
Kuwait	0.831	Very high HDI	2.13	2.58E-06	0.56	1.69E-06	4.64	6.15E-06	-	-	-	-	-	-
Brunei Darussalam	0.829	Very high HDI	1.94	7.33E-06	0.42	4.20E-06	3.63	1.48E-05	-	-	-	-	-	-
Russian Federation	0.822	Very high HDI	3.54	6.03E-07	1.39	5.41E-07	5.74	1.09E-06	0.01	2.67E-08	0.01	3.78E-08	0.01	3.78E-08
Romania	0.821	Very high HDI	2.69	1.55E-06	1.09	1.37E-06	4.41	2.85E-06	-	-	-	-	-	-
Oman	0.816	Very high HDI	1.93	1.80E-06	0.68	1.17E-06	4.89	5.43E-06	0.05	2.50E-07	0.03	2.12E-07	0.10	7.07E-07
Bahamas	0.812	Very high HDI	1.96	8.00E-06	4.00	1.63E-05	-	-	-	-	-	-	-	-
Kazakhstan	0.811	Very high HDI	0.86	7.79E-07	0.24	6.00E-07	1.48	1.44E-06	0.01	1.00E-07	-	-	0.01	1.00E-07
Trinidad and Tobago	0.810	Very high HDI	0.93	2.80E-06	-	-	1.87	5.64E-06	-	-	-	-	-	-
Costa Rica	0.809	Very high HDI	5.83	3.76E-06	1.39	2.58E-06	10.39	7.14E-06	-	-	-	-	-	-
Uruguay	0.809	Very high HDI	4.67	4.41E-06	0.91	2.74E-06	8.52	8.48E-06	-	-	--	-	-	-
Belarus	0.808	Very high HDI	5.07	2.85E-06	2.19	2.66E-06	8.09	5.13E-06	-	-	-	-	-	-
Panama	0.805	Very high HDI	3.22	3.14E-06	0.67	2.02E-06	5.81	5.99E-06	0.03	3.00E-07	-	-	0.06	6.00E-07

Malaysia	0.803	Very high HDI	1.10	6.30E-07	0.48	5.78E-07	1.77	1.15E-06	0.03	1.00E-07	0.01	7.07E-08	0.06	2.27E-07
Georgia	0.802	Very high HDI	2.25	2.95E-06	-	-	4.58	6.01E-06	-	-	-	-	-	-
Mauritius	0.802	Very high HDI	0.86	3.04E-06	0.63	3.64E-06	1.09	4.87E-06	-	-	-	-	-	-
Serbia	0.802	Very high HDI	0.25	6.68E-07	0.05	5.00E-07	0.47	1.30E-06	-	-	-	-	-	-
Thailand	0.800	Very high HDI	1.47	5.77E-07	0.40	4.26E-07	2.55	1.08E-06	0.03	8.66E-08	0.01	5.77E-08	0.04	1.33E-07
Albania	0.796	High HDI	0.49	1.55E-06	0.37	1.85E-06	0.62	2.53E-06	-	-	-	-	-	-
Bulgaria	0.795	High HDI	2.23	2.31E-06	0.55	1.53E-06	4.03	4.51E-06	-	-	-	-	-	-
Sri Lanka	0.782	High HDI	3.96	1.68E-06	1.13	1.30E-06	6.52	2.98E-06	0.15	3.27E-07	0.08	3.58E-07	0.22	5.50E-07
Bosnia Herzegovina	0.780	High HDI	1.24	2.43E-06	0.46	2.06E-06	2.05	4.47E-06	-	-	-	-	-	-
Iran, Islamic Republic of Iran	0.774	High HDI	1.75	4.91E-07	0.59	3.96E-07	2.91	8.99E-07	0.03	6.88E-08	0.03	1.06E-07	0.03	9.05E-08
Ukraine	0.773	High HDI	2.89	9.92E-07	1.12	8.69E-07	4.74	1.81E-06	0.02	7.56E-08	0.03	1.34E-07	0.02	1.41E-07
China	0.768	High HDI	14.30	3.73E-07	8.77	4.05E-07	20.32	6.41E-07	0.06	2.47E-08	0.04	2.90E-08	0.08	4.00E-08
Moldova	0.767	High HDI	4.49	3.84E-06	3.12	4.46E-06	5.91	6.30E-06	-	-	-	-	-	-
Dominican Republic	0.767	High HDI	1.67	1.41E-06	0.94	1.51E-06	2.39	2.37E-06	-	-	-	-	-	-
Cuba	0.764	High HDI	2.68	1.94E-06	0.27	9.00E-07	5.22	3.87E-06	0.01	1.00E-07	0.02	2.00E-07	-	-
Peru	0.762	High HDI	2.19	9.25E-07	0.85	7.93E-07	3.49	1.65E-06	0.05	1.39E-07	0.02	1.15E-07	0.07	2.21E-07
Mexico	0.758	High HDI	3.41	5.84E-07	1.59	5.66E-07	5.18	1.01E-06	0.02	3.92E-08	0.02	6.03E-08	0.03	7.75E-08
Brazil	0.754	High HDI	4.99	5.42E-07	1.18	3.66E-07	8.83	1.02E-06	0.02	3.38E-08	0.02	4.85E-08	0.02	4.71E-08
Colombia	0.752	High HDI	2.80	8.37E-07	0.73	6.02E-07	4.89	1.57E-06	0.03	8.66E-08	0.02	1.00E-07	0.04	1.41E-07
Maldives	0.747	High HDI	1.37	4.57E-06	0.57	3.29E-06	3.15	1.29E-05	0.41	2.37E-06	0.57	3.29E-06	-	-
Turkmenistan	0.745	High HDI	0.55	1.06E-06	0.24	9.80E-07	3.84	8.38E-06	0.12	4.90E-07	0.24	9.80E-07	-	-
Algeria	0.745	High HDI	2.19	7.92E-07	0.58	5.74E-07	1.08	4.19E-07	0.11	1.91E-07	0.04	1.51E-07	0.17	3.33E-07
Azerbaijan	0.745	High HDI	0.88	1.03E-06	0.69	1.28E-06	0.87	1.31E-06	0.05	2.89E-07	-	-	0.10	5.77E-07
Ecuador	0.740	High HDI	3.43	1.57E-06	0.67	9.77E-07	6.23	3.00E-06	0.03	1.50E-07	0.01	1.00E-07	0.04	2.31E-07
Mongolia	0.739	High HDI	0.41	1.18E-06	0.06	6.00E-07	0.75	2.26E-06	-	-	-	-	-	-
Tunisia	0.731	High HDI	1.03	1.07E-06	0.35	9.04E-07	1.71	1.95E-06	0.15	4.16E-07	0.30	8.32E-07	-	-
Egypt	0.731	High HDI	0.85	3.27E-07	0.37	3.01E-07	1.34	5.85E-07	0.02	5.55E-08	0.01	7.07E-08	0.03	9.05E-08
Fiji	0.730	High HDI	2.80	6.42E-06	-	-	5.71	1.31E-05	-	-	-	-	-	-

Suriname	0.730	High HDI	1.87	6.61E-06	-	-	3.83	1.35E-05	-	-	-	-	-	-
Uzbekistan	0.727	High HDI	0.41	3.91E-07	0.21	3.90E-07	0.61	6.78E-07	0.01	5.77E-08	0.01	1.00E-07	0.01	7.07E-08
Jordan	0.720	High HDI	2.73	1.83E-06	0.67	1.27E-06	4.83	3.47E-06	0.05	2.50E-07	-	-	0.10	5.00E-07
Libya	0.718	High HDI	0.92	1.28E-06	0.24	9.07E-07	1.61	2.40E-06	-	-	-	-	-	-
Paraguay	0.717	High HDI	2.81	2.16E-06	0.72	1.54E-06	4.98	4.11E-06	0.01	1.00E-07	0.03	3.00E-07	-	-
Gaza Strip and West Bank	0.715	High HDI	2.85	2.62E-06	0.99	2.16E-06	4.77	4.84E-06	0.15	6.12E-07	0.05	5.00E-07	0.26	1.16E-06
Saint Lucia	0.715	High HDI	2.52	1.26E-05	-	-	4.96	2.48E-05	-	-	-	-	-	-
South Africa	0.713	High HDI	0.68	3.63E-07	0.20	2.77E-07	1.17	6.78E-07	0.01	4.47E-08	-	-	0.02	1.00E-07
Samoa	0.707	High HDI	3.76	1.68E-05	-		8.01	3.58E-05	-	-	-	-	-	-
Lebanon	0.706	High HDI	2.37	2.12E-06	1.31	2.28E-06	3.35	3.49E-06	0.05	2.89E-07	0.04	4.00E-07	0.07	4.95E-07
Gabon	0.706	High HDI	0.21	1.05E-06	-		0.42	2.10E-06	-	-	-	-	-	-
Indonesia	0.705	High HDI	2.11	3.20E-07	0.90	2.92E-07	3.35	5.74E-07	0.08	6.15E-08	0.04	6.32E-08	0.13	1.14E-07
Viet Nam	0.703	High HDI	2.52	5.66E-07	0.90	4.78E-07	4.21	1.04E-06	0.10	1.11E-07	0.05	1.21E-07	0.16	2.00E-07
Philippines	0.699	Medium HDI	2.37	5.19E-07	0.76	4.13E-07	4.03	9.65E-07	0.04	6.58E-08	0.02	6.67E-08	0.06	1.13E-07
Botswana	0.693	Medium HDI	0.11	7.78E-07	-		0.21	1.48E-06	-	-	-	-	-	-
Bolivia	0.692	Medium HDI	0.63	8.27E-07	0.16	6.05E-07	1.11	1.55E-06	0.04	2.31E-07	0.07	4.04E-07	-	-
Kyrgyzstan	0.692	Medium HDI	0.55	1.02E-06	0.34	1.13E-06	0.76	1.70E-06		-	-	-	-	-
Venezuela	0.691	Medium HDI	1.33	7.92E-07	0.45	6.56E-07	2.18	1.42E-06	0.02	1.00E-07	0.02	1.41E-07	0.02	1.41E-07
Iraq	0.686	Medium HDI	2.43	8.71E-07	0.92	7.51E-07	4.01	1.60E-06	0.10	1.74E-07	0.05	1.77E-07	0.16	3.20E-07
Tajikistan	0.685	Medium HDI	0.07	3.13E-07	0.02	2.00E-07	0.12	6.00E-07	-	-	-	-	-	-
Morocco	0.683	Medium HDI	2.72	9.68E-07	0.58	6.29E-07	4.86	1.83E-06	0.05	1.29E-07	0.01	7.07E-08	0.09	2.50E-07
El Salvador	0.675	Medium HDI	0.02	2.00E-07	0.05	5.00E-07	-		-	-	-	-	-	-
Nicaragua	0.667	Medium HDI	1.93	1.87E-06	0.71	1.59E-06	3.18	3.43E-06	0.05	2.89E-07	-	-	0.11	6.35E-07
Bhutan	0.666	Medium HDI	0.13	1.30E-06	-		0.28	2.80E-06	-	-	-	-	-	-
Cape Verde	0.662	Medium HDI	0.24	2.40E-06	-		0.49	4.90E-06	-	-	-	-	-	-
Bangladesh	0.661	Medium HDI	0.36	1.61E-07	0.16	1.52E-07	0.56	2.85E-07	0.02	3.92E-08	0.01	3.54E-08	0.03	7.07E-08
India	0.633	Medium HDI	0.67	7.67E-08	0.26	6.63E-08	1.13	1.45E-07	0.05	2.12E-08	0.03	2.26E-08	0.07	3.59E-08
Ghana	0.632	Medium HDI	0.15	2.43E-07	0.23	4.27E-07	0.07	2.33E-07	0.03	1.13E-07	0.04	1.79E-07	0.02	1.41E-07

Guatemala	0.627	Medium HDI	1.05	8.41E-07	0.89	1.10E-06	1.20	1.26E-06	0.03	1.34E-07	0.03	2.12E-07	0.04	2.31E-07
Honduras	0.621	Medium HDI	1.79	1.46E-06	0.35	9.04E-07	3.25	2.79E-06	0.02	1.41E-07	0.05	3.54E-07	-	-
Namibia	0.615	Medium HDI	0.43	1.43E-06	0.09	9.00E-07	0.76	2.69E-06	0.05	5.00E-07	-	-	0.10	1.00E-06
Lao People's Democratic Republic	0.607	Medium HDI	2.48	2.02E-06	0.89	1.71E-06	4.09	3.67E-06	0.10	4.08E-07	0.03	3.00E-07	0.16	7.16E-07
Vanuatu	0.607	Medium HDI	3.00	1.13E-05	-	-	5.84	2.21E-05	-	-	-	-	-	-
Timor-Leste	0.607	Medium HDI	0.57	2.33E-06	0.18	1.80E-06	0.96	4.29E-06	-	-	-	-	-	-
Nepal	0.602	Medium HDI	0.96	6.28E-07	1.08	1.03E-06	0.90	8.05E-07	0.08	1.84E-07	0.09	3.00E-07	0.07	2.21E-07
Equatorial Guinea	0.596	Medium HDI	0.21	1.21E-06	-	-	0.57	3.29E-06	-	-	-	-	-	-
Cambodia	0.593	Medium HDI	1.87	1.15E-06	0.67	9.99E-07	3.06	2.06E-06	0.05	1.89E-07	0.03	2.12E-07	0.07	3.13E-07
Zimbabwe	0.593	Medium HDI	0.07	2.47E-07	0.04	2.83E-07	0.10	4.08E-07	-	-	-	-	-	-
Angola	0.586	Medium HDI	0.09	1.92E-07	0.06	2.45E-07	0.13	3.25E-07	-	-	-	-	-	-
Myanmar	0.585	Medium HDI	1.47	5.87E-07	0.57	5.20E-07	2.35	1.04E-06	0.05	1.07E-07	0.02	8.94E-08	0.08	1.94E-07
Syrian Arab Republic	0.577	Medium HDI	2.72	1.30E-06	1.89	1.53E-06	3.57	2.13E-06	0.11	2.52E-07	0.12	3.79E-07	0.10	3.33E-07
Cameroon	0.576	Medium HDI	0.13	2.55E-07	-	-	0.26	5.10E-07	0.02	1.00E-07	-	-	0.04	2.00E-07
Kenya	0.575	Medium HDI	0.19	2.10E-07	0.04	1.41E-07	0.34	3.95E-07	0.01	5.77E-08	-	-	0.01	5.77E-08
Zambia	0.565	Medium HDI	0.05	1.77E-07	0.05	2.50E-07	0.06	3.00E-07	-	-	-	-	-	-
Solomon Islands	0.564	Medium HDI	2.67	7.41E-06	-	-	5.40	1.50E-05	0.22	2.20E-06	-	-	0.43	4.30E-06
Papua New Guinea	0.558	Medium HDI	1.99	1.69E-06	0.31	9.35E-07	3.75	3.31E-06	0.11	3.89E-07	-	-	0.23	8.13E-07
Mauritania	0.556	Medium HDI	0.22	7.78E-07	0.17	9.81E-07	0.28	1.25E-06	-	-	-	-	-	-
Côte d'Ivoire	0.550	Medium HDI	0.23	3.35E-07	0.11	3.32E-07	0.36	6.00E-07	0.01	1.00E-07	-	-	0.01	1.00E-07
Tanzania, United Republic of Tanzania	0.549	Low HDI	0.13	1.65E-07	0.05	1.44E-07	0.21	2.97E-07	0.01	4.47E-08	-	-	0.02	8.94E-08
Pakistan	0.544	Low HDI	0.78	2.10E-07	0.42	2.15E-07	1.16	3.66E-07	0.06	6.03E-08	0.04	6.32E-08	0.07	9.11E-08
Togo	0.539	Low HDI	0.19	5.48E-07	0.04	4.00E-07	0.35	1.06E-06	-	-	-	-	-	-
Haiti	0.535	Low HDI	0.64	8.33E-07	0.52	1.06E-06	0.76	1.28E-06	0.03	1.73E-07	0.05	3.54E-07	0.02	2.00E-07
Nigeria	0.535	Low HDI	0.07	6.53E-08	-	-	0.15	1.40E-07	0.01	3.33E-08	-	-	0.01	3.33E-08
Rwanda	0.534	Low HDI	0.27	5.10E-07	0.10	4.47E-07	0.43	8.97E-07	0.01	1.00E-07	-	-	0.02	2.00E-07
Uganda	0.525	Low HDI	0.24	2.67E-07	0.13	2.84E-07	0.35	4.52E-07	0.02	7.07E-08	0.01	7.07E-08	0.03	1.22E-07

Benin	0.525	Low HDI	0.11	3.48E-07	-	-	0.22	6.96E-07	-	-	-	-	-	-
Malawi	0.512	Low HDI	0.07	2.02E-07	0.03	2.12E-07	0.12	3.79E-07	0.01	1.00E-07	-	-	0.01	1.00E-07
Senegal	0.511	Low HDI	0.20	4.08E-07	0.15	5.00E-07	0.24	6.20E-07	-	-	-	-	-	-
Djibouti	0.509	Low HDI	0.62	2.77E-06	-	-	1.34	5.99E-06	-	-	-	-	-	-
Sudan	0.508	Low HDI	0.23	2.62E-07	0.15	2.83E-07	0.30	4.29E-07	0.01	7.07E-08	0.01	1.00E-07	0.01	1.00E-07
Madagascar	0.501	Low HDI	0.20	3.02E-07	0.10	3.02E-07	0.29	5.05E-07	0.01	7.07E-08	-	-	0.02	1.41E-07
The Republic of the Gambia	0.500	Low HDI	0.29	1.30E-06	0.25	1.77E-06	0.34	1.96E-06	-	-	-	-	-	-
Ethiopia	0.498	Low HDI	1.28	3.72E-07	0.55	3.44E-07	2.02	6.63E-07	0.12	1.11E-07	0.05	1.04E-07	0.19	1.97E-07
Eritrea	0.492	Low HDI	0.76	1.66E-06	0.37	1.65E-06	1.15	2.87E-06	0.10	5.77E-07		-	0.21	1.21E-06
Liberia	0.481	Low HDI	0.14	6.26E-07	0.06	6.00E-07	0.22	1.10E-06	-	-	-	-	-	-
Congo, Democratic People Republic of Congo	0.479	Low HDI	0.08	1.13E-07	0.05	1.21E-07	0.10	1.74E-07	0.01	4.08E-08	0.01	7.07E-08	0.01	5.00E-08
Afghanistan	0.478	Low HDI	0.41	3.64E-07	0.22	3.77E-07	0.63	6.53E-07	0.04	1.03E-07	0.04	1.51E-07	0.05	1.77E-07
Sierra Leone	0.477	Low HDI	0.04	2.83E-07	0.04	4.00E-07	0.03	3.00E-07	-	-	-	-	-	-
Guinea	0.465	Low HDI	0.12	3.62E-07	0.08	4.00E-07	0.15	5.67E-07	0.03	2.12E-07	-	-	0.05	3.54E-07
Yemen	0.455	Low HDI	1.00	6.31E-07	0.32	4.94E-07	1.69	1.17E-06	0.09	1.88E-07	0.02	1.15E-07	0.16	3.58E-07
Mozambique	0.446	Low HDI	0.32	3.62E-07	0.18	3.93E-07	0.46	6.09E-07	0.06	1.55E-07	0.02	1.41E-07	0.10	2.77E-07
Mali	0.428	Low HDI	0.33	4.97E-07	0.19	5.27E-07	0.46	8.26E-07	0.02	1.41E-07	-	-	0.03	2.12E-07
Burundi	0.426	Low HDI	0.17	4.25E-07	0.04	2.83E-07	0.30	8.02E-07	0.04	2.00E-07	-	-	0.08	4.00E-07
Central African Republic	0.404	Low HDI	0.08	4.62E-07	0.04	4.00E-07	0.12	8.49E-07	-	-	-	-	-	-
Niger	0.400	Low HDI	0.32	4.48E-07	0.12	4.00E-07	0.52	8.02E-07	0.02	1.15E-07	-	-	0.04	2.31E-07
Chad	0.394	Low HDI	1.37	1.15E-06	0.25	6.68E-07	2.50	2.22E-06	0.33	5.58E-07	0.07	3.50E-07	0.60	1.08E-06
South Sudan	0.385	Low HDI	0.32	6.16E-07	0.12	5.37E-07	0.52	1.11E-06	0.06	2.68E-07	-	-	0.12	5.37E-07

Note: HDI, Human Development Index. ASR, age-standardized rate per 100,000 population. ASIR, age-standardized incidence rate. ASMR, age-standardized mortality rate. SE, standard error.

Supplementary Table 3. National EAPC for ASIR of early-onset thyroid cancer by sex during the available and unified periods.

Countries(periods)	Available period		Unified period (2001-2010)	
	Males	Females	Males	Females
Republic of Korea(1999-2012)	25.81	20.42	29.95	23.04
Turkey(1998-2012)	15.37	13.52	18.65	15.49
Poland(1998-2012)	11.70	7.48	1.99	2.12
Chile(1998-2012)	10.27	9.67	-14.76	9.32
China(1988-2012)	10.16	6.24	17.01	10.57
Ireland(1994-2012)	8.49	9.42	9.33	7.23
Lithuania(1978-2012)	8.16	6.31	9.51	4.56
Ukraine(2003-2015)	7.90	7.39	4.72	4.74
Croatia(1988-2012)	6.88	6.92	9.17	8.03
Belarus(1978-2012)	6.03	6.92	-1.29	0.88
Bulgaria(1993-2013)	5.90	4.78	7.55	6.79
Brazil(1993-2012)	5.70	7.61	2.25	4.88
Italy(1988-2012)	5.62	7.23	5.16	5.88
Slovenia(1963-2012)	5.60	6.41	0.04	5.32
India(1983-2012)	5.44	4.41	2.47	3.51
Malta(1992-2013)	5.43	1.98	-2.04	-1.76
Czechia(1983-2013)	5.36	5.99	7.41	7.75
Latvia(1983-2012)	4.94	6.09	-1.86	9.30
Slovakia(1971-2010)	4.93	5.76	7.52	8.79
Canada(1983-2012)	4.60	5.33	4.46	3.28
UK, England and Wales(1971-2017)	4.52	4.91	6.21	6.55
Ecuador(1985-2012)	4.49	7.26	6.48	9.74
Colombia(1983-2012)	4.48	3.50	1.05	5.37
France(1988-2012)	4.40	5.27	0.60	2.85
Costa Rica(1982-2011)	4.05	5.43	11.32	7.16
Australia(1982-2016)	3.99	3.82	1.94	2.93
Japan(1988-2010)	3.95	2.49	5.92	8.35
Denmark(1953-2016)	3.32	4.03	6.63	2.76
UK, Scotland(1975-2013)	3.25	3.23	4.22	4.17
New Zealand(1983-2012)	2.95	3.25	4.45	5.04
Estonia(1968-2012)	2.54	3.65	26.30	0.86
Spain(1993-2010)	2.52	2.69	5.31	4.71
The Netherlands(1989-2013)	2.43	2.95	7.72	2.92
USA: White(1975-2016)	2.38	3.37	4.56	5.39
Switzerland(1998-2012)	2.29	5.87	3.81	5.56
USA(1975-2016)	2.21	3.19	4.42	5.02
Norway(1953-2016)	2.12	1.98	1.92	5.17
Thailand(1993-2012)	2.00	1.78	-4.59	-1.26
Israel(1963-2012)	1.79	2.85	8.33	3.70
USA: Black(1975-2016)	1.75	2.72	3.89	5.45

Germany(1998-2012)	1.44	7.19	2.88	7.55
Sweden(1960-2016)	1.38	1.74	5.67	5.39
Iceland(1955-2016)	1.17	0.96	-16.57	-4.84
UK, Northern Ireland(1993-2013)	0.54	3.37	4.17	7.44
Uganda(1991-2015)	-0.20	-2.54	-21.60	9.29
Kuwait: Kuwaiti(1998-2012)	-1.58	5.82	-4.84	4.35

Note: EAPC, Estimated Annual Percentage Change.

Supplementary Table 4. Global EAPC for ASIR and ASMR of early-onset cancer at different sites by sex during the unified period.

Cancer types	EAPC in ASIR (2001-2010)		EAPC in ASMR (2000-2014)	
	Males	Females	Males	Females
Thyroid	9.8819	9.2843	-0.3828	-1.3348
Corpus uteri	-	4.1295	-	2.5903
Prostate	3.9521	-	0.6913	-
Kidney	2.9925	2.4731	-0.9273	-1.8378
Colon	2.6612	3.3615	0.3233	-0.1015
Colorectum	2.5885	3.4195	0.3903	0.2765
Rectum and anus	2.4874	3.5172	0.5272	1.1221
Multiple myeloma	1.8740	1.3758	-1.1178	-1.8829
Testis	1.8209	-	0.0855	-
Pancreas	1.6271	3.5281	-1.1823	-0.7180
NHL	1.4071	1.2261	-3.4168	-3.2485
Uterus	-	1.3951	-	-0.5779
Hodgkin lymphoma	1.1353	0.9256	-2.7705	-3.6459
Cervix uteri	-	1.0202	-	-0.2039
Breast	-	0.8382	-	-1.5896
Leukemia	0.7246	0.7996	-2.1070	-2.0509
Brain, CNS	0.6650	0.6709	-0.2576	-0.3977
Lip, oral cavity and pharynx	0.6268	1.6954	-1.8284	-0.8872
Gallbladder	0.6178	-0.7092	-1.3972	-1.9145
Esophagus	0.4763	-0.8230	-1.6670	-0.3814
Melanoma of skin	0.3671	0.5099	-1.9177	-1.9037
Bladder	0.0397	-1.1582	-1.4468	-0.5166
Liver	0.0036	0.3656	-2.7516	-1.0086
Ovary	-	-0.0558	-	-0.8004
Lung	-0.4664	0.5914	-3.5148	-3.3202
Larynx	-1.7059	-1.4546	-4.6985	-3.0949
Stomach	-2.2914	-0.7369	-2.0989	-1.6604

Note: EAPC, Estimated Annual Percentage Change.

Supplementary Table 5. Global ASIR and ASMR of early-onset thyroid cancer by sex during the unified period.

Year	ASIR (2001-2010)		ASMR (2000-2014)	
	Males	Females	Males	Females
2000	-	-	0.012	0.018
2001	0.827	3.775	0.012	0.015
2002	0.906	4.218	0.013	0.016
2003	0.970	4.605	0.013	0.012
2004	0.998	5.102	0.014	0.013
2005	1.128	5.239	0.013	0.015
2006	1.138	5.615	0.013	0.016
2007	1.408	6.237	0.012	0.011
2008	1.531	7.107	0.014	0.012
2009	1.755	8.013	0.013	0.011
2010	1.847	8.377	0.012	0.017
2011	-	-	0.013	0.012
2012	-	-	0.013	0.013
2013	-	-	0.012	0.014
2014	-	-	0.012	0.013

Note: ASIR, age-standardized incidence rate per 100,000 population. ASMR, age-standardized mortality rate per 100,000 population.

Supplementary Table 6. National EAPC for ASMR of early-onset thyroid cancer by sex during the available and unified periods.

Countries(periods)	Available period		Unified period (2000-2014)	
	Males	Females	Males	Females
Costa Rica(1997-2017)	7.62	-0.32	7.77	5.40
Slovenia(1985-2018)	6.72	2.57	2.32	38.53
Belgium(1992-2016)	5.04	-2.85	6.10	-2.53
Argentina(1997-2018)	3.56	-1.56	-1.87	1.42
Chile(1997-2018)	2.47	2.06	9.62	3.26
Brazil(1996-2018)	2.08	1.05	1.47	-1.36
Mexico(1998-2017)	0.79	2.39	0.92	3.52
Republic of Korea(1995-2018)	0.76	-3.35	8.00	-6.53
Spain(1955-2017)	0.19	-1.23	-9.48	-4.22
Ecuador(1997-2017)	0.55	1.41	0.19	-0.57
Malta(1978-2017)	-5.5511E-14	-10.25	0	0
Singapore(1966-2017)	-0.31	-5.06	10.00	5.57
New Zealand(1955-2016)	-0.36	-2.10	-6.21	4.11
UK, Northern Ireland (1955-2016)	-0.73	-11.48	0	0
USA: Black(1973-2017)	-0.83	-1.47	4.43	1.60
USA: White(1973-2017)	-0.87	0.15	-2.26	-0.23
Hungary(1970-2018)	-0.88	-3.28	0.61	30.17
Israel(1975-2018)	-0.94	-5.14	-4.35	6.99
Australia(1955-2018)	-1.00	-2.42	-5.62	0.01
UK, England and Wales (1955-2016)	-1.18	-2.51	0.62	-0.14
Romania(1999-2018)	-1.47	-6.80	1.56	-1.69
Sweden(1955-2018)	-1.51	-3.26	9.08	-5.95
The Netherlands(1955-2018)	-1.52	-2.55	-4.33	-8.06
France(1955-2016)	-1.56	-2.67	-2.70	-6.12
USA(1955-2017)	-1.58	-2.21	-1.29	-0.09
Switzerland(1955-2017)	-1.69	-4.34	-7.78	-11.42
Japan(1955-2018)	-1.80	-2.79	-0.49	-1.78
Kyrgyzstan(2000-2016)	-1.97	-6.63	-3.91	-11.41
United Kingdom(1967-2016)	-1.99	-3.28	-2.19	-0.99
Croatia(1985-2017)	-2.04	2.54	-1.78	-14.30
Canada(1955-2017)	-2.11	-2.54	1.89	-0.30
Poland(1999-2018)	-2.13	-5.33	1.08	-4.13
Latvia(1996-2018)	-2.17	-31.46	-5.07	0
Greece(1966-2017)	-2.38	-2.91	-3.44	-2.57
Bulgaria(1966-2018)	-2.45	-1.26	-7.34	-4.90
Italy(1969-2017)	-2.60	-4.59	1.67	-1.04
UK, Scotland(1955-2018)	-2.66	-4.22	-21.96	-5.81
Denmark(1955-2018)	-2.80	-2.18	4.05	-11.98
Germany(1983-2018)	-2.81	-3.46	-0.80	-2.59
Austria(1955-2018)	-3.09	-4.17	-9.24	-16.28

Finland(1955-2018)	-3.19	-3.17	-30.72	5.80
Ireland(1955-2015)	-3.24	-2.37	2.64	16.29
Colombia(1997-2017)	-3.35	2.23	0.30	-0.33
Cuba(2000-2017)	-3.40	10.21	-2.31	4.58
Portugal(1984-2018)	-3.92	-1.59	-4.74	-3.36
Czechia(1986-2018)	-4.83	-8.00	0.48	2.43
Lithuania(1993-2018)	-6.96	0.02	-0.67	-32.92
Slovakia(1992-2014)	-7.21	3.56	-13.48	-2.63
Uruguay(1997-2017)	-15.56	-2.04	-19.66	2.20

Note: EAPC, Estimated Annual Percentage Change.

Supplementary Table 7. Projected cases and deaths (of thousands) from early-onset thyroid cancer by HDI and sex from 2022 to 2050.

Year	Cases								Deaths							
	Low HDI countries		Medium HDI countries		High HDI countries		Very high HDI countries		Low HDI countries		Medium HDI countries		High HDI countries		Very high HDI countries	
	Males	Females	Males	Females	Males	Females	Males	Females	Males	Females	Males	Females	Males	Females	Males	Females
2022	0.92	3.01	2.92	11.77	51.47	122.35	8.78	37.59	0.09	0.27	0.24	0.54	0.34	0.73	0.07	0.10
2025	1.00	3.32	3.07	12.36	53.21	122.81	8.75	37.52	0.09	0.30	0.25	0.56	0.35	0.74	0.07	0.10
2030	1.13	3.77	3.24	12.93	46.82	107.14	8.52	36.54	0.11	0.34	0.26	0.58	0.32	0.68	0.07	0.09
2035	1.26	4.21	3.37	13.26	44.26	100.27	8.30	35.67	0.12	0.38	0.26	0.59	0.31	0.65	0.07	0.09
2040	1.39	4.66	3.43	13.40	44.53	100.12	8.14	35.15	0.13	0.42	0.27	0.60	0.31	0.65	0.06	0.09
2045	1.51	5.08	3.40	13.34	44.08	99.58	8.04	34.75	0.14	0.46	0.26	0.59	0.31	0.65	0.07	0.09
2050	1.61	5.47	3.34	13.23	40.03	92.56	7.80	33.80	0.14	0.50	0.26	0.58	0.29	0.62	0.07	1.00

Note: HDI, Human Development Index.
